# Supplementary material for: The Influence of Vitamin D Status on Cognitive Ability in Patients with Bipolar Disorder and Healthy Controls
Source: Nutrients. 2023 Sep 22;15(19):4111. doi: 10.3390/nu15194111 (PMC10574501; doi:10.3390/nu15194111)
Supplement: Supplementary file 1 [file nutrients-15-04111-s001.zip › nutrients-2592687-supplementary.pdf]

nutrients

MDPI

Supplementary Materials

The Influence of Vitamin D Status on Cognitive Ability in Patients with Bipolar Disorder and Healthy Controls

S1. Hierarchical Regression Analyses in Healthy Controls

S1.1. Attention

The results of the multiple hierarchical regression analysis indicated no significant association between the individuals' levels of 25(OH)D, 24,25(OH)2D3 or VMR and "attention". In all steps (Model 1: $F(1, 91) = 13.41$, $p < 0.001$; Model 2: $F(2, 90) = 14.06$, $p < 0.001$; Model 3: $F(3, 89) = 9.65$, $p < 0.001$; Model 4: $F(4, 88) = 7.17$, $p < 0.001$, Model 5: $F(5, 87) = 5.70$, $p < 0.001$), age showed a significant effect on "attention", as well as premorbid IQ once it was taken in the model (see Table S1).

S1.2. Memory

Age showed a significant effect on memory over all steps of the multiple hierarchical regression analysis. Premorbid IQ showed a significant effect on memory once it was taken in the model (Model 1: $F(1, 91) = 21.76$, $p < 0.001$; Model 2: $F(2, 90) = 15.39$, $p < 0.001$; Model 3: $F(3, 89) = 10.55$, $p < 0.001$; Model 4: $F(4, 88) = 7.82$, $p < 0.001$; Model 5: $F(5, 87) = 6.28$, $p < 0.001$). No significant associations between 25(OH)D, 24,25(OH)2D3 or VMR and memory were found (see Table S1).

S1.3. Executive Function

The results of the multiple hierarchical regression analysis indicated no significant association between 25(OH)D, 24,25(OH)2D3 or VMR and "executive function". In all steps (Model 1: $F(1, 91) = 23.26$, $p < 0.001$; Model 2: $F(2, 90) = 23.76$, $p < 0.001$; Model 3: $F(3, 89) = 15.67$, $p < 0.001$; Model 4: $F(4, 88) = 11.63$, $p < 0.001$, Model 5: $F(5, 87) = 9.20$, $p < 0.001$), age showed a significant effect on "attention", as well as premorbid IQ once it was taken in the model (see Table S1).

Table S1. Association of age, premorbid IQ, 25(OH)D, 24,25(OH)2D3, VMR with "attention", "memory", and "executive function" in healthy controls.

		Attention			Memory			Executive Function		
		β	*t*	*p*	β	*t*	*p*	β	*t*	*p*
Model 1	Age	−0.36	−3.66	**<0.001**	−0.44	−4.67	**<0.001**	−0.45	−4.82	**<0.001**
Model 2	Age	−0.46	−4.79	**<0.001**	−0.52	−5.43	**<0.001**	−0.57	−6.37	**<0.001**
	Premorbid IQ	0.35	3.60	**<0.001**	0.26	2.73	**0.008**	0.40	4.42	**<0.001**
Model 3	Age	−0.50	−4.79	**<0.001**	−0.55	−5.38	**<0.001**	−0.57	−5.84	**<0.001**
	Premorbid IQ	0.35	3.66	**<0.001**	0.27	2.80	**0.006**	0.39	4.38	**<0.001**
	25(OH)D	0.09	0.94	0.350	0.09	0.95	0.347	−0.01	−0.09	0.932
Model 4	Age	−0.50	−4.77	**<0.001**	−0.55	−5.35	**<0.001**	−0.57	−5.81	**<0.001**
	Premorbid IQ	0.36	3.64	**<0.001**	0.27	2.76	**0.007**	0.40	4.34	**<0.001**
	25(OH)D	0.08	0.60	0.553	0.09	0.73	0.471	−0.02	−0.16	0.872
	$24,25(OH)_2D_3$	0.03	0.22	0.828	0.01	0.02	0.988	0.02	0.15	0.879
Model 5	Age	−0.50	−4.73	**<0.001**	−0.56	−5.35	**<0.001**	−0.57	−5.77	**<0.001**
	Premorbid IQ	0.36	3.63	**<0.001**	0.26	2.68	**0.009**	0.40	4.30	**<0.001**
	25(OH)D	0.07	0.53	0.597	0.11	0.81	0.419	−0.02	−0.17	0.865
	$24,25(OH)_2D_3$	0.05	0.35	0.728	−0.05	−0.31	0.758	0.02	0.17	0.868
	VMR	−0.04	−0.31	0.759	0.07	0.59	0.557	−0.01	−0.07	0.944

Note: Attention: Model 1: $R^2 = 0.13$, R^2corr = 0.12, Model 2: $R^2 = 0.24$, R^2corr = 0.22, Model 3: $R^2 = 0.25$, R^2corr = 0.22, Model 4: $R^2 = 0.25$, R^2corr = 0.21, Model 5: $R^2 = 0.25$, R^2corr = 0.20; Memory: Model 1: $R^2 = 0.19$, R^2corr = 0.18, Model 2: $R^2 = 0.26$, R^2corr = 0.24, Model 3: $R^2 = 0.26$, R^2corr = 0.24, Model 4: $R^2 = 0.26$, R^2corr = 0.23, Model 5: $R^2 = 0.27$, R^2corr = 0.22; Executive Function: Model 1: $R^2 = 0.20$, R^2corr = 0.20, Model 2: $R^2 = 0.35$, R^2corr = 0.33, Model 3: $R^2 = 0.35$, R^2corr = 0.32, Model 4: $R^2 = 0.35$, R^2corr = 0.32, Model 5: $R^2 = 0.35$, R^2corr = 0.31. Bold printed *p*-values are significant.
